# The mediating effects of self-directed learning ability and critical thinking ability on the relationship between learning engagement and problem-solving ability among nursing students in Southern China: a cross-sectional study

**DOI:** 10.1186/s12912-023-01280-2

**Published:** 2023-06-19

**Authors:** Lingling Huang, Xuanhua Li, Ya Meng, Ming Lei, Yanru Niu, Shanshan Wang, Rong Li

**Affiliations:** 1 School of Nursing, Lida University, Shanghai, 201609 China; 2 Department of Nursing, School of Medical, Huanghe Science and Technology College, Zhengzhou, 450000 China; 3 Department of Nursing, School of Nursing, Philippine Women’s University, Manila, Philippines; 4 Shanghai General Hospital, Shanghai, 200080 China

**Keywords:** Learning engagement, Self-directed learning ability, Critical thinking ability, Problem-solving ability, Nursing students

## Abstract

**Background:**

Problem-solving ability has been identified as a core competence that nursing students should develop, and it plays a vital role in career development. Therefore, it is necessary to investigate factors related to problem-solving ability and the path relationships among those factors in the context of nursing students.

**Objective:**

This study aims to identify the factors that affect problem-solving ability, and to investigate path relationships of self-directed learning ability, critical thinking ability, learning engagement, and problem-solving ability among nursing students.

**Design:**

A cross-sectional study.

**Settings:**

The Department of Nursing at a university located in Shanghai, China.

**Sample:**

A total of 540 nursing students with a three-year education program were enrolled in the current study.

**Methods:**

Data were collected by using a structured questionnaire, including general information, learning engagement, self-directed learning ability, critical thinking ability, and problem-solving ability of nursing students. Pearson’s correlations were used to explore the relationships between learning engagement, self-directed learning ability, critical thinking ability, and problem-solving ability. The path relationships were analyzed by constructing a structural equation model using AMOS software.

**Results:**

Our results showed that learning engagement, self-directed learning ability, and critical thinking ability were positively associated with problem-solving ability. Furthermore, learning engagement did not influence problem-solving ability directly, but it affected problem-solving ability indirectly via self-directed learning ability and critical thinking ability among nursing students. Additionally, the total effects of self-directed learning (0.442) and critical thinking ability (0.581) were more prominent than learning engagement (0.361) on problem-solving ability.

**Conclusions:**

To improve the problem-solving ability of nursing students, nursing educators should develop targeted strategies to enhance learning engagement, self-directed learning ability, and critical thinking ability.

## Introduction

People are facing increasingly complicated health problems, and their demands for medical and health care are becoming more diversified [1]. People expect personalised and high quality health care, which requires nurses to have not only basic medical knowledge, but also specific core competencies. Problem-solving ability is one of these core competencies. Problem-solving ability involves cognitive, emotional, and behavioural activities [2], which is a process that identifies a situation as a problem, selects appropriate measures, and implements these measures to achieve the expected goals [3]. Previous studies have shown that nurses with strong problem-solving ability can deal with patients’ health problems and emergencies more effectively, and can even reduce disease recurrence and mortality [5, 6]. In other words, problem-solving ability is crucial for nurses to make clinical reasoning and decisions [4], and nurses with strong problem-solving ability are able to evaluate patients thoroughly, identify health problems immediately, and provide holistic care [5].

However, previous studies reported that nursing students’ problem-solving ability was still at a medium or low level [6–8]. When recently graduated nursing students enter the clinic, they lack rational thinking and decision-making ability. Moreover, it is difficult for them to use existing resources and practical methods to analyze and solve clinical problems, resulting in low efficiency in terms of nursing work, and even the emergence of negative emotions, which will have a negative effect on their career [9]. Therefore, problem-solving ability should be cultivated among nursing students immediately. And it is necessary to identify the factors related to problem-solving ability and to investigate the relationship among these factors.

The engagement was initially defined as a positive, fulfilling, and work-related mental state characterized by vitality, dedication, and absorption [10]. More recently, the scope of this concept shifted from a sole focus on work to encompass learning. Learning engagement is defined as a students’ ongoing efforts to achieve learning outcomes in the learning process, which is a multidimensional phenomenon that includes emotional engagement (i.e. enthusiasm and interest), cognitive engagement (i.e. the use of learning strategies and self-regulation), and behavioural engagement (i.e. effort, persistence, and attention) [11, 12]. Nursing students’ learning engagement is regarded as a primary component of effective teaching and a necessary prerequisite for learning [13]. Additionally, learning engagement is also a key component in nursing education, and it significantly predicts nursing students’ learning achievements and personal development [14–16]. Liu et al. noted that students with higher levels of emotional and behavioural engagement have stronger self-directed learning ability [17]. Accordingly, learning engagement was positively associated with self-directed learning ability. However, to the best of our knowledge, few researchers have examined the correlations between learning engagement and critical thinking ability or problem-solving ability.

Due to the rapid technological advances in improving healthcare and living standards, nursing students need to constantly keep up to date with the latest evidence-based practice and knowledge. Self-directed learning ability is the most crucial factor in facilitating personal learning with the aim of providing efficient medical and care services to patients [18]. This ability focuses on learning needs and goals, making individuals responsible for their learning and helping individuals actively and independently seek appropriate methods to solve problems [19]. Nursing students who learn knowledge and skills on their own can effectively seek, analyze, and use the information to solve problems [20]. Previous studies have found a significant positive correlation between self-directed learning ability and problem-solving ability, and reported that self-directed learning ability could affect problem-solving ability directly among first and second-year nursing students in South Korea [13]. Similarly, a cross-sectional study conducted among junior or senior nursing students in South Korea showed that the development and implementation of appropriate self-directed learning programmes are critical for improving nursing students’ problem-solving ability [21]. Moreover, Choi et al. demonstrated that the higher independent learning scores of first-year nursing students were, the better their problem-solving and critical thinking ability would be [22]. These results indicated that self-directed learning ability played a key role in determining problem-solving ability and critical thinking ability among nursing students.

Critical thinking ability in nursing professionals is defined as the ability to think, apply, analyze, synthesize, and evaluate situations [23]. The ability refers to a complex mental process that helps nursing students make decisions and take action when facing unexpected nursing situations after recognizing, synthesizing, analyzing, and evaluating the relevant information [20, 24]. It allows individuals to raise questions logically, understand situations, and criticize solutions to problems [23]. Studies on the relationship between critical thinking ability and problem-solving ability have shown that an improvement in an individual’s critical thinking ability can lead to improvement in an individual’s problem-solving ability [25]. And Jo suggested that in order to improve the problem-solving ability of undergraduate nursing students, it is necessary to develop their critical thinking ability [26].

In summary, although previous studies have explored the importance of problem-solving ability in nursing education, as well as the association between problem-solving ability and certain other important competencies among nursing students, such as self-directed learning ability and critical thinking ability, the evidence remained insufficient, because the results that have previously been reported were inconsistent in terms of the populations studied due to populations from different regions, different education programmes, and different grades. Furthermore, according to the literature review just conducted, few studies have directly focused on the associations of learning engagement with critical thinking ability or problem-solving ability, but some studies have emphasized the association of learning engagement with self-directed learning ability, the association of problem-solving ability with self-directed learning ability and critical thinking ability Therefore, a structural equation model of problem-solving ability was hypothesized and established. This study aims to explore the structural model of the relationships between learning engagement, self-directed learning ability, critical thinking, and problem-solving ability among three-year nursing students in Southern China. And it will provide the necessary foundational data to support the design of educational programs aimed at improving the problem-solving ability of nursing students.

## Methods

### Materials and methods

#### Study design and participants

A cross-sectional survey was conducted between March and April 2022, in the Department of Nursing with a three-year education program at a university in Shanghai, China. The survey samples were selected using a convenience sampling method. First, we selected one university from a list of all universities with a department of nursing featuring a three-year education program by drawing lots. Subsequently, we chose some freshman, sophomore, and junior classes. Finally, all nursing students from the selected classes completed the structured questionnaires. Students were included if they had a normal mental status. The exclusion criteria were as follows:1) refusal to participate in this study (n = 3); 2) missing or invalid data on learning engagement, self-directed learning ability, critical thinking, and problem-solving ability variables (n = 7). A total of 540 eligible participants were recruited for the final analysis. The response rate was 98.12%.

#### Procedures

All research data were collected using structured questionnaires. In order to improve data collection skills and standardize data collection methods, the interviewers received specific training in advance. The project leader informed the interviewers of certain communication skills with nursing students, and explained the purpose and method of filling in the questionnaire in advance.

### Measurements

#### College students’ learning engagement questionnaire

The 20-item learning engagement questionnaire [27]. with a Cronbach’s alpha of 0.85 was applied to assess the level of college students’ learning engagement, including behavioural engagement (6 items), cognitive engagement (7 items), and emotional engagement (7 items). Participants reported their learning engagement level by responding to items scored on a 5-point scale (1–5), which ranged from “1 = completely nonconformity” to “5 = completely conformity”, and higher scores indicated higher levels of learning engagement. The Cronbach’s alpha was 0.961 in this study.

#### Nursing students’ self-directed learning scale

The nursing students’ self-directed learning scale consisted of 20 items with a Cronbach’s alpha of 0.914, including learning motivation (6 items), planning and implementation (6 items), self-management (4 items), and interpersonal communication (4 items). Each item was scored on a 5-point scale (1–5), which ranged from “1 = strongly disagree” to “7 = strongly agree”, and higher scores indicated higher self-directed learning ability levels [28, 29]. The Cronbach’s alpha was 0.965 in this study.

#### Critical thinking disposition inventory

Nursing students’ critical thinking ability was measured using the Critical Thinking Disposition Inventory-Chinese Version (CIDI-CV) [30]. This scale was composed of 7 subscales with truth seeking (10 items), open mindedness (10 items), analyticity (10 items), systematicity (10 items), self-confidence (10 items), inquisitiveness (10 items), and maturity (10 items). Each item was scored on a 6-point scale (1–6), which ranged from “1 = strongly disagree” to “6 = strongly agree”. Higher scores on this scale indicated higher levels of critical thinking ability. The Cronbach’s alpha was 0.90 in Peng’s study. The Cronbach’s alpha was 0.947 in this study.

#### Social problem-solving inventory

Nursing students’ social problem-solving ability was assessed using the Chinese Version of the Social Problem-Solving Inventory (C-SPSI), which was validated based on the Social Problem-Solving Inventory Revised (SPSI-R) [31]. This scale was composed of the 5 subscales of the 25-item SPSI-R, including positive problem orientation (5 items), rational problem solving (5 items), negative problem orientation (5 items), impulsivity/carelessness style (4 items), and avoidance style (6 items). Each item was scored on a 5-point scale (1–5); the first two subscales ranged from “1 = completely nonconformity” to “5 = completely conformity”, but the last three subscales ranged from “1 = completely conformity” to “5 = completely nonconformity”. Higher scores on this scale indicated higher levels of problem-solving ability. Cronbach’s alpha was 0.817 in Siu’s study. The Cronbach’s alpha was 0.997 in this study.

### Statistics analysis

Data analyses were performed using SPSS V.21.0 and AMOS V24.0 software. Data are presented as the mean ± SD, frequencies (percentage), and correlation coefficient. Pearson’s correlation was used to explore the relationships between learning engagement, self-directed learning ability, critical thinking ability, and problem-solving ability among nursing students. Hypotheses were examined by conducting a structural equation modeling analysis. The model was tested using the goodness of fit test. In this context, the most important measures are the goodness of fit index (GFI), the adjusted goodness of fit index (AGFI), the incremental fit index (IFI), the tucker-lewis coefficient (TLI), the comparative fitting index (CFI), the root mean square error of approximation (RMSEA), and the chi-square degrees of freedom (x2/df). A two-tailed P < 0.05.was regarded as statistical significance.

## Results

### Participants’ general information

A total of 540 participants were included in our study, and their mean age was 20.18 ± 1.29 years. Most participants were female (87.2%), only children (66.9%), and more than half of the students lived in a city. 60.6% loved nursing, and 65.9% stayed in a general learning atmosphere. More details of the participants’ characteristics were presented in Table 1.

**Table 1:** General information of nursing students (*N* = 540)

Variable	Categories	N (%) or Mean ± SD
Gender	Male	69(12.8)
	Female	471(87.2)
Age (year)		20.18 ± 1.29
Grade	Freshman	193(35.7)
	Sophomore	175(32.4)
	Junior	172(31.9)
Home adress		
	City	369(69.3)
	Countryside	171(31.7)
Only children		
	Yes	361(66.9)
	No	179(33.1)
Student leader		
	Yes	109(20.2)
	No	431(79.8)
Learning atmosphere in the classrom		
	Strong	176(32.6)
	General	356(65.9)
	Poor	8(1.5)
Love of nursing		
	Srong	207(38.3)
	General	327(60.6)
	Poor	6(1.1)

### Descriptive statistics of measured variables

The average scores of nursing students’ learning engagement, self-directed learning ability, critical thinking ability, and problem-solving ability were 71.27 ± 11.85, 71.34 ± 11.35, 269.16 ± 27.46, and 81.74 ± 8.81, respectively (shown in Table 2). The mean scores of learning engagement and self-directed learning ability were at the upper middle, indicating that the students in this study were willing to explore knowledge actively, and made efforts to achieve learning outcomes in their learning process; The mean scores of critical thinking ability and problem-solving ability were at the middle level, indicating that relevant educators need to pay more attention on improving critical thinking ability and problem-solving ability of the students in this study.

**Table 2:** Level of learning engagement, self-directed learning ability, critical thinking ability and problem-solving ability among nursing students (*N* = 540)

Variable	Mean ± SD	Min-Max
**learning engagement**	71.27 ± 11.85	20–100
behavioural engagement	21.27 ± 3.75	6–31
cognitive engagement	25.37 ± 4.45	7–35
emotional engagement	24.62 ± 4.46	7–35
**Self-directed learning ability**	71.34 ± 11.35	20–100
learning motivation	21.69 ± 3.81	6–30
planning and implementation	21.27 ± 3.68	6–30
self-management	14.10 ± 2.43	4–20
Interpersonal communication	14.28 ± 2.48	4–20
**Critical thinking ability**	269.16 ± 27.46	218–388
truth seeking	30.38 ± 6.28	9–50
open mindedness	38.94 ± 5.56	24–60
analyticity	41.11 ± 4.82	25–60
systematicity	38.06 ± 5.37	27–60
self-confidence	40.05 ± 5.58	24–60
inquisitiveness	43.59 ± 6.36	28–60
maturity	37.03 ± 7.22	10–59
**Problem-solving ability**	81.74 ± 8,81	57–109
positive problem orientation	17.72 ± 3.01	5–25
rational problem solving	17.70 ± 3.01	5–25
negative problem orientation	14.57 ± 3.47	5–25
impulsivity/carelessness style	11.74 ± 2.71	4–20
avoidance style	15.34 ± 4.74	6–30

### Correlation between measured variables

Table 3 showed significant positive correlations between learning engagement and self-directed learning ability (r = 0.817, P < 0.001), between learning engagement and critical thinking ability (r = 0.383, P < 0.001), between learning engagement and problem-solving ability (r = 0.326, P < 0.001), between self-directed learning ability and critical thinking ability (r = 0.527, P < 0.001), between self-directed learning ability and problem-solving ability (r = 0.442, P < 0.001), and between critical thinking ability and problem-solving ability (r = 0.652, P < 0.001).

**Table 3:** Correlation beween learning engagement, self-directed learning ability, critical thinking ability and problem-solving ability among nursing students (*N* = 540)

Variable	Learning engagement	Self-directed learning ability	Critical thinking ability	Problem-solving ability
	r(p)			
Learning engagement	1			
Self-directed learning ability	0.817(< 0.001)	1		
Critical thinking ability	0.383(< 0.001)	0.527(< 0.001)	1	
Problem-solving ability	0.326(< 0.001)	0.442(< 0.001)	0.652(< 0.001)	1

### Structural equation model test

The theoretical model indexes were χ2/df < 5, GFI > 0.9, AGFI > 0.9, IFI > 0.9, TLI > 0.9, CFI > 0.9, and RMSEA < 0.08 [32]. The model fitness indexes in this study were χ2/df = 2.609, GFI = 0.995, AGFI = 0.976, IFI = 0,997, TLI = 0.991, CFI = 0.997, and RMSEA = 0.055, which all met the theoretical values. Therefore, the hypothesized path model was appropriate. The fitness indices were shown in Table 4.

**Table 4:** Structure equation modeling fit indices

Fitness	χ2/df	GFI	AGFI	IFI	TLI	CFI	RMSEA
Acceptable values	< 3	> 0.9	> 0.9	> 0.9	> 0.9	> 0.9	< 0.08
Mediation model	2.609	0.995	0.976	0.997	0.991	0.997	0.055

GFI— goodness of fifit index; AGFI—Adjusted Goodness of Fit Index; IFI—incremental fit index; TLI—tucker-lewis coefficien; CFI—comparative fifit index; RMSEA—root mean square error of approximation

### Effects of structural equation model

**Fig. 1:**
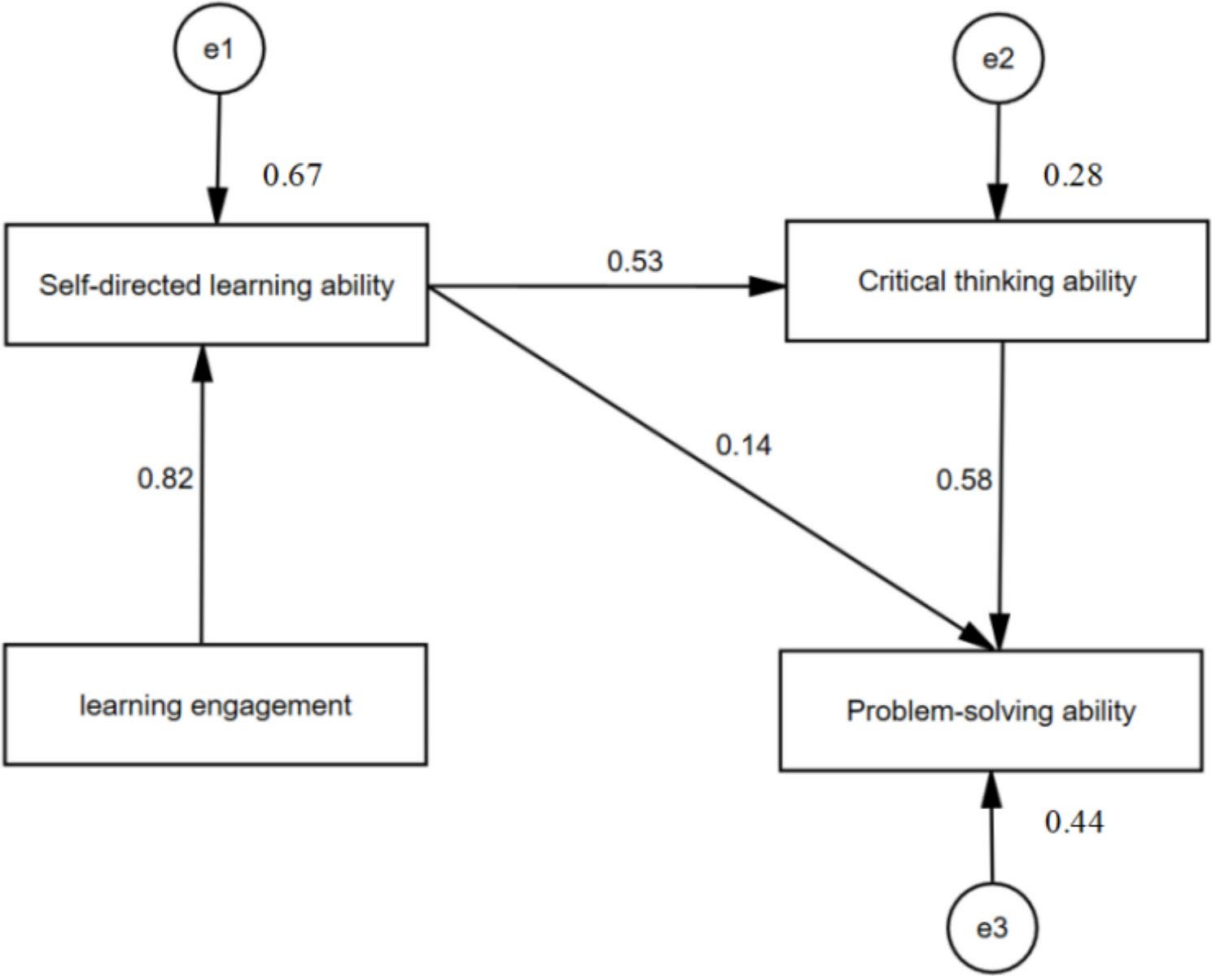
The mediating effects of self-directed learning ability and critical thinking ability on the relationship between learning engagement and problem-solving ability

The standardized direct, indirect, and total effects of the variables included in the structural equation model were provided in Table 5. Learning engagement had a direct effect on self-directed learning ability (0.817). Self-directed learning ability had a direct effect on critical thinking ability (0.527). However, learning engagement had no direct effect on critical thinking ability. Self-directed learning ability had a direct effect on problem-solving ability (0.136), and critical thinking ability had a direct effect on problem-solving ability (0.581), but learning engagement had no direct effect on problem-solving ability. In other words, self-directed learning ability (0,817*0.136 = 0.111) and critical thinking ability (0.817*0,527*0.581 = 0.250) mediated the relationship between the nursing students’ learning engagement and problem-solving ability. Although nursing students’ learning engagement did not affect their problem-solving ability directly, high levels of learning engagement improved their problem-solving ability by enhancing their self-directed learning ability and critical thinking ability. The total effect of learning engagement on problem-solving ability was 0.361 (0,817*0.136 + 0.817*0,527*0.581). The path diagram for the model was presented in Fig. 1.

**Table 5:** Standardized direct, indirect, and total effects of each variable in structural equation model

Independent variable	Dependent variable	Direct effect	Indirect effect	Total effect
Learning engagement	Self-directed learning ability	0.817		0.817
Self-directed learning ability	Critical thinking ability	0.527		0.527
Self-directed learning ability	Problem-solving ability	0.136	0.306	0.442
Critical thinking ability	Problem-solving ability	0.581		0.581
Learning engagement	Critical thinking ability		0.431	0.431
Learning engagement	Problem-solving ability		0.361	0.361

## Discussion

### Main findings

Our results showed that learning engagement, self-directed learning ability, and critical thinking ability were significantly positively associated with the problem-solving ability of nursing students. Furthermore, learning engagement did not influence problem-solving ability directly, but it affected problem-solving ability indirectly via self-directed learning ability and critical thinking ability. Additionally, our results confirmed that the total effects of both self-directed learning ability and critical thinking ability on problem-solving ability were more prominent than that of learning engagement on problem-solving ability.

### The positive association of learning engagement, self-directed learning ability, and critical thinking ability with problem-solving ability

In this study, the problem-solving ability was significantly positively related to learning engagement (r = 0.326, P < 0.001), self-directed learning ability (r = 0.442, P < 0.001), and critical thinking ability (r = 0.652, P < 0.001). These findings indicate that the higher the level of learning engagement, self-directed learning ability, and critical thinking ability of nursing students is, the higher their problem-solving ability will be. In other words, the problem-solving ability can be improved by enhancing learning engagement, self-directed learning ability, and critical thinking ability, similar to previous findings [13, 22, 33, 34]. Students with high levels of learning engagement are more likely to exhibit strong enthusiasm and desire for knowledge, and they would do their best to complete challenging tasks [35]; therefore, such students are more willing to seek problem-solving methods. Concerning the association of problem-solving ability with self-directed learning ability, our findings were consistent with that of previous studies [13, 22]. Nursing students with strong self-directed learning ability are more responsible for their learning, and can effectively seek, analyze, and use the information to solve problems [19, 20]. Fang et al. found that self-directed learning ability might be recommended as an effective way to enhance the level of problem-solving ability of Chinese baccalaureate nursing students [36]. Regarding the relationship between problem-solving ability and critical thinking ability, the results in this study were similar to Kanbay’s reports [25]. It may be that individuals with greater critical thinking have stronger confidence in solving a problem, which in turn enhances their problem-solving ability [37]. Therefore, it is necessary for educators to develop teaching methods to facilitate nursing students’ learning engagement, self-directed learning ability, and critical thinking ability for improving nursing students’ problem-solving ability.

### The mediating effects of self-directed learning and critical thinking ability on the relationship between learning engagement and problem-solving ability

In the current study, learning engagement was not found to have a significant direct effect on critical thinking ability and problem-solving ability, but had a mediating effect on problem-solving ability through self-directed learning ability and critical thinking ability. An explanation for these findings was that learning engagement directly and positively influences self-directed learning ability. Emotional and behavioural engagement were associated with self-directed learning ability [17]. Students with high levels of emotional engagement exhibited stronger interest and confidence in learning, had a relatively correct understanding of the value of learning, and were more likely to learn actively and independently; Students with high levels of behavioural engagement were more serious and focused, and when they encountered difficulties in learning, they were more willing to ask teachers or classmates for help to solve problems. Hence, they had stronger self-directed learning ability [38]. The mediating effects highlighted the need to consider learning engagement, self-directed learning ability, and critical thinking ability in nursing students’ education when identifying factors related to problem-solving ability. Moreover, a significant finding in our study was that the total effects of self-directed learning (0.442) and critical thinking ability (0.581) were more prominent than learning engagement (0.361) on problem-solving ability among nursing students enrolled in a three-year education program. These results emphasize the need for educators to consider self-directed learning and critical thinking ability together when they focus on using learning engagement to improve problem-solving ability. Educators should adopt teaching strategies that emphasize the development of self-directed learning and critical thinking ability to improve problem-solving ability, such as flipped learning, team-based learning (TBL) and problem-based learning (PBL). Cheng et al. [39] and Qamata et al. [40] demonstrated that undergraduate nursing students’ self-directed learning ability could be significantly improved by TBL and PBL approaches. A quasi-experimental research conducted in Borujen Nursing School suggested nursing students’ critical thinking ability could be enhanced by the school training course [41].

### Strengths and limitations of our study

To the best of our knowledge, the mediating effects of self-directed learning ability and critical thinking ability on the relationship between learning engagement and problem-solving ability were explored for the first time, especially in nursing students enrolled in a three-year education program in Southern China. Improving nursing students’ problem-solving ability is very necessary, because previous studies have suggested that nurses with high levels of problem-solving ability were more capable of dealing with patients’ problems [2]. However, this study has several limitations. First, the data in our analyses were based on self-reports, which could lead to biases or inaccuracies. Second, this study was a cross-sectional design, so the observed associations should not be assumed to be causal relationships. Further in-depth studies with longitudinal follow-up data are warranted to explore the cause-effect relationship. Third, this study was performed only in Shanghai, China, and the generalization of the results should be carefully promoted.

## Conclusions

This study showed that self-directed learning ability and critical thinking ability affected nursing students’ problem-solving ability directly, and the association of learning engagement with problem-solving ability was influenced by the mediating effects of self-directed learning ability and critical thinking ability. Therefore, it is necessary for educators to develop improvement strategies for nursing students’ problem-solving ability in consideration of these variables.

## Data Availability

As the data are shared by our team, the datasets generated and/or analysed during the current study are not publicly available, but are available from the corresponding author on reasonable request.
